# Targeted metabolomics reveals fatty acid abundance adjustments as playing a crucial role in drought-stress response and post-drought recovery in wheat

**DOI:** 10.3389/fgene.2022.972696

**Published:** 2022-11-10

**Authors:** Safi Ullah, Mudassar Nawaz Khan, Sumaira Salahuddin Lodhi, Iftikhar Ahmed, Muhammad Tayyab, Tariq Mehmood, Israr Ud Din, Majid Khan, Quahir Sohail, Muhammad Akram

**Affiliations:** ^1^ Institute of Biotechnology and Genetic Engineering, The University of Agriculture Peshawar, Peshawar, Pakistan; ^2^ Department of Biotechnology and Genetic Engineering, Hazara University Mansehra, Mansehra, Pakistan; ^3^ Department of Biochemistry, Hazara University Mansehra, Mansehra, Pakistan; ^4^ National Culture Collection of Pakistan, Land Resources Research Institute, National Agricultural Research Centre, Islamabad, Pakistan; ^5^ Department of Agriculture, Hazara University Mansehra, Mansehra, Pakistan; ^6^ AgroBioSiences, University Mohammed VI Polytechnic (UM6P), Ben Guerir, Morocco; ^7^ Medicinal Botanic Centre, PCSIR Labs Complex Peshawar, Peshawar, Pakistan

**Keywords:** wheat, drought, Atta Habib, metabolomics, fatty acids, recovery

## Abstract

Drought stress is one of the abiotic stresses restricting plant development, reproductive growth, and survival. In the present study, the effect of drought stress and post-drought recovery for the selected local wheat cultivar, Atta Habib, was studied. Wheat was grown for 16 days followed by drought stress for 7 days and allowed to recover for 7 days after the removal of the drought stress. Same-aged untreated plants were also grown as a control. The effect of drought stress and post-drought recovery on morphology (root length, shoot length, root weight, and shoot weight), enzymatic activity, and fatty acid profile were analyzed. The results showed that shoot weight (93.1 mg), root weight (85.2 mg), and shoot length (11.1 cm) decreased in the stressed plants but increased steadily in the recovered plants compared to the same-aged control plants, while root length showed a higher increase (14.0 cm) during drought stress and tended to normalize during the recovery phase (13.4 cm). The ascorbate peroxidase activity increased in the stressed plants (5.44 unit/mg protein) compared to the control, while gradually normalizing in the recovery phase (5.41 unit/mg protein). Gas chromatography coupled mass spectrometric analysis revealed abundance changes in important fatty acids, such as palmitic acid, stearic acid, oleic acid, linoleic acid, and linolenic acid. Palmitic acid (39.1%) and oleic acid (2.11%) increased in the drought-stressed plants, while a reduction in linoleic acid (6.85%) and linolenic acid (51.18%) was observed compared to the same-aged control plants, i.e., palmitic (33.71%), oleic (0.95%), linoleic (7.52%), and linolenic acid (55.23%). The results suggest that wheat tries to recover in the post-drought stage by repairing oxidative damage through ascorbate peroxidase, and by adjusting fatty acid abundances under drought stress and during the post-drought phase in an effort to maintain membranes’ integrity and a suitable fat metabolism route, thus helping recovery. Targeted metabolomics may be further used to explore the role of other metabolites in the drought-stress response mechanism in wheat. Furthermore, this relatively little explored avenue of post-drought recovery needs more detailed studies involving multiple stress durations.

## Introduction

Wheat (*Triticum aestivum* L.) is attracting worldwide attention due to the rising global population and comprehensive environmental changes (Lucas et al., 2011). Wheat, a greatest energy source, is composed of 58.2% starch, and also it has an adequate amount of body fats and sugar. The worldwide wheat production in 2020–21 was 778.60 million metric tons, with major producers being China, India, Russia, the United States, Australia, France, Canada, and Ukraine (USDA, 2021). Leading wheat consumers include China, the European Union, India, Russia, the United States, and Pakistan (USDA, 2021). In Pakistan, wheat is used as a staple food and accounts for 8.7% of value addition in agriculture and 1.7% of GDP. In 2020–21, wheat production in Pakistan was recorded as 27.293 million metric tons according to the Pakistan Economic Survey 2021 released by the Ministry of Finance (PES, 2021).

Climatic changes have led to abiotic stresses such as drought being more frequent and severe for a number of important crops, such as rice, maize, cotton, tea, sorghum, soybean, and wheat (Farooq et al., 2009). Global climatic changes have led to an increase in the global temperature over the years, which has led to the aggravation of drought events (Lal et al., 2021). Drought restricts plants’ reproductive growth, development, and survival. Drought is associated with a water-supply restriction throughout the reproductive, growth, and developmental stages (Flexas and Medrano, 2002). More than 70% of fertile land around the globe is affected by drought, and the yield loss related to drought stress has gained much attention in recent years. Drought decreases the leaf size and the number of leaves per plant, as well as reducing the leaf longevity (Shao et al., 2008). Drought exerts many physiological effects on plants, including decreased photosynthetic activity (Qureshi et al., 2007), increased oxidative stress, altered cell-wall elasticity (Caruso et al., 2009), abscisic acid accumulation, and toxic metabolite generation (Ahuja et al., 2010). Plants accumulate biomolecules such as proline and melatonin (Tiwari et al., 2021). The most essential organ of a plant is its roots, which have the ability to search for, and supply water to, the plant (Hawes et al., 2000). It is the first organ to be affected by water-limiting stress (Shimazaki et al., 2005). Roots continue to develop to find water in the drought-stress state, but the aerial organs of the plant are restricted in their growth.

The stress responses of plants signify a highly dynamic and complex method seeking to establish a unique homeostasis under adverse growth conditions. The mechanisms that are drought-sensitive include fatty acid metabolism, amino acid metabolism, modulation of cell structure, regulation of gene expression, scavenging of oxygen-reactive species, synthesis of osmolyte, nitrogen assimilation, metabolism of energy and carbohydrate, induction of hormones, kinase cascade signaling, and ion channels activation (Wang et al., 2016). The improvement of the water-deficit tolerance in crops has emerged as a key challenge for today’s plant scientists (Budak et al., 2013). Plants recognize and respond to stress conditions with a variety of biological signals at appropriate times and speeds for their survival (Takahashi and Shinozaki, 2019). Higher plants achieve sophisticated responses and adaptations to abiotic stresses, including drought, to maintain optimal growth under stress conditions. For these complex physiological responses in plants, a variety of cellular and molecular regulatory mechanisms are required for short-term responses to prevent water loss *via* transpiration from guard cells and for long-term adaptations to acquire stress resistance at the whole-plant level (Nakashima et al., 2014; Takahashi et al., 2018).

Targeted metabolomics, through utilizing powerful techniques such gas chromatography-mass spectrometry (GC-MS), is an outstanding analytical method for providing valuable separation and resolution. The combination of excellent recognition and separation of the GC-MS technique facilitates a comparatively balanced analysis of a number of known and unknown metabolites (Desbrosses et al., 2005).

Fatty acids play vital roles in the membrane structure of lipids in all living cells. These hydrophobic compounds can also play particular roles in signaling events and metabolic processes. Triacylglycerol is a lipid that is a common type of high-energy compound for storage in many organisms, including plants, where it is present in the seeds of many species (Graham and Eastmond, 2002). In all plant cells, the glycerol-lipid structural membranes include almost entirely 16-carbon and 18-carbon fatty acids, which usually have three methylene-interrupted double bonds (16:0, 16:1*, 18:0, 18:1, 18:2, 18:3, and in some species 16:3) (Ruiz-Lopez et al., 2015). It is equally important to analyze the post-drought recovery stage to reveal valuable information regarding the mechanisms that occur in plants to recover from drought stress. Several oxidative stress-related proteins, including superoxide dismutase, oxidoreductase, and aldehyde reductase, have been found to increase in the roots of *Vigna radiata* in response to drought stress and recovery (Sengupta and Reddy, 2011). In the present study, in order to investigate the response of wheat to drought stress and post-drought recovery, morphological, enzymatic, and GC-MS-based metabolite analyses are performed.

## Materials and methods

### Plant growth and treatment

**FIGURE 1:**
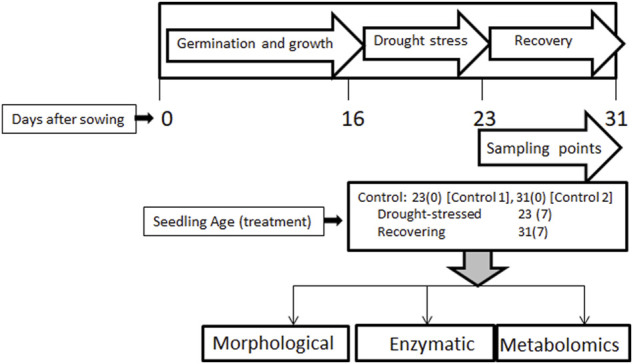
The experimental design of the study showing wheat germination, drought-stress application, post-drought recovery stages, and types of analyses performed. Wheat seeds were sown and grown till 16 days. Drought stress was applied on these seedlings for 7 days, followed by 7 days of recovery. Age-matched control plants were also sampled, along with drought-stressed and post-drought recovery plants. 23(0) and 31(0) represent control-1 and control-2 plants, whereas 23(7) and 31(7) represent the drought-stressed and post-drought recovery plants after 7 days of drought stress, respectively.

Seeds of a wheat cultivar, Atta Habib, were sown in small pots in a greenhouse at the Institute of Biotechnology & Genetic Engineering, University of Agriculture, Peshawar. About 7–8 seeds per pot, with 16 pots per replication, were sown. Sampling was performed for three replications for morphological, enzymatic, and metabolomic analyses. After 16 days of sowing, drought stress was applied for 7 days while keeping some as a control. Stress was removed after 7 days, and some of the stressed pots were kept for the recovery phase for 7 days. Two kinds of samples were collected at the end of stress period, i.e., one control (control-1) and one stressed (after 23 days), and two kinds of samples were collected after 31 days, i.e., control-2 and the post-drought recovery sample (Figure 1).

### Morphological analysis

The weights and lengths of the shoots and roots were measured. For this purpose, the roots were taken with extreme care in order to avoid root damage. Soil was removed from roots by washing with tap water. At each time point collection, this same process was repeated.

### Ascorbate peroxidase assay

A weight of 200 mg of the sample was homogenized in 2.5 ml of 25 mM potassium phosphate buffer (pH 7.8) containing 2% polyvinyl-pyrrolidone, 0.4 mM EDTA, and 1 mM ascorbic acid. The solution was centrifuged at 15,000 g for 20 min at 4°C; the clear supernatant was collected to measure APX activity. Protein content was measured by Bradford assay (Bradford, 1976), using bovine serum albumin as the standard. For the measurement of APX activity, the reaction mixture comprised 25 mM potassium phosphate buffer (pH 7.0), 0.25 mM ascorbic acid, 0.4 mM EDTA, and 0.1 mM H_2_O_2_. APX activity was determined following the depletion in absorbance at 290 nm using a UV/Vis spectrophotometer (Nakano and Asada, 1987).

### Fatty acid analysis using gas chromatography-mass spectrometry (GC-MS)

Fatty acid composition was analyzed using GC-MS (PERKIN ELMER SQ8S). For the extraction and preparation of the standard and samples, the protocol described by Katoch (2011) was used. For fatty acids extraction, weights of 120 mg of the plant sample and the powdered standard form were put in glass vials. 1 ml of petroleum spirit was added in each and Polytron was used for crushing. Ethanol was used to rinse the Polytron between the samples. The samples were centrifuged at ambient temperature for 5 min at 5,000 rpm. A clear supernatant of 1 ml after centrifugation was transferred for transmetylation in another tube. In the next step, 0.5 ml of sodium methylated solution (10 g of CH_3_ONa in 500 ml methanol) was added, and this was incubated for 30 min at room temperature to complete the transmetylation reaction. In the last step of sample preparation, 0.5 ml 1 M NaCl solution was added, and after 5 min the mixture was divided into two separate layers. The upper layer was used for further fatty acid analysis. The following conditions of GC-MS were maintained: column temperature, 120°C–200°C; injector temperature, 250°C; and ion source temperature, 200°C. GC-MS analysis was programmed for 45.67 min. Peaks of the fatty acid methyl esters were identified through the NIST mass spectral library and comparing their retention time with that of the known standards run under similar separation conditions.

### Statistical analysis

One-way ANOVA was applied for analyzing data variations among groups at different analyzed time points, followed by Tukey’s HSD post-hoc test, utilizing the SPSS software package.

## Results

Seeds of a wheat cultivar; Atta Habib, were sown in small pots in a greenhouse. After 16 days of sowing, stress was applied for 7 days while keeping some as a control. Stress was removed after 7 days and some of the stressed pots were kept for the recovery phase for 7 days. Three types of analyses, i.e., morphological, enzymatic, and GC-MS based metabolomics, were performed for fatty acid profiling.

### Effect of drought stress on the Atta Habib phenotype

**FIGURE 2:**
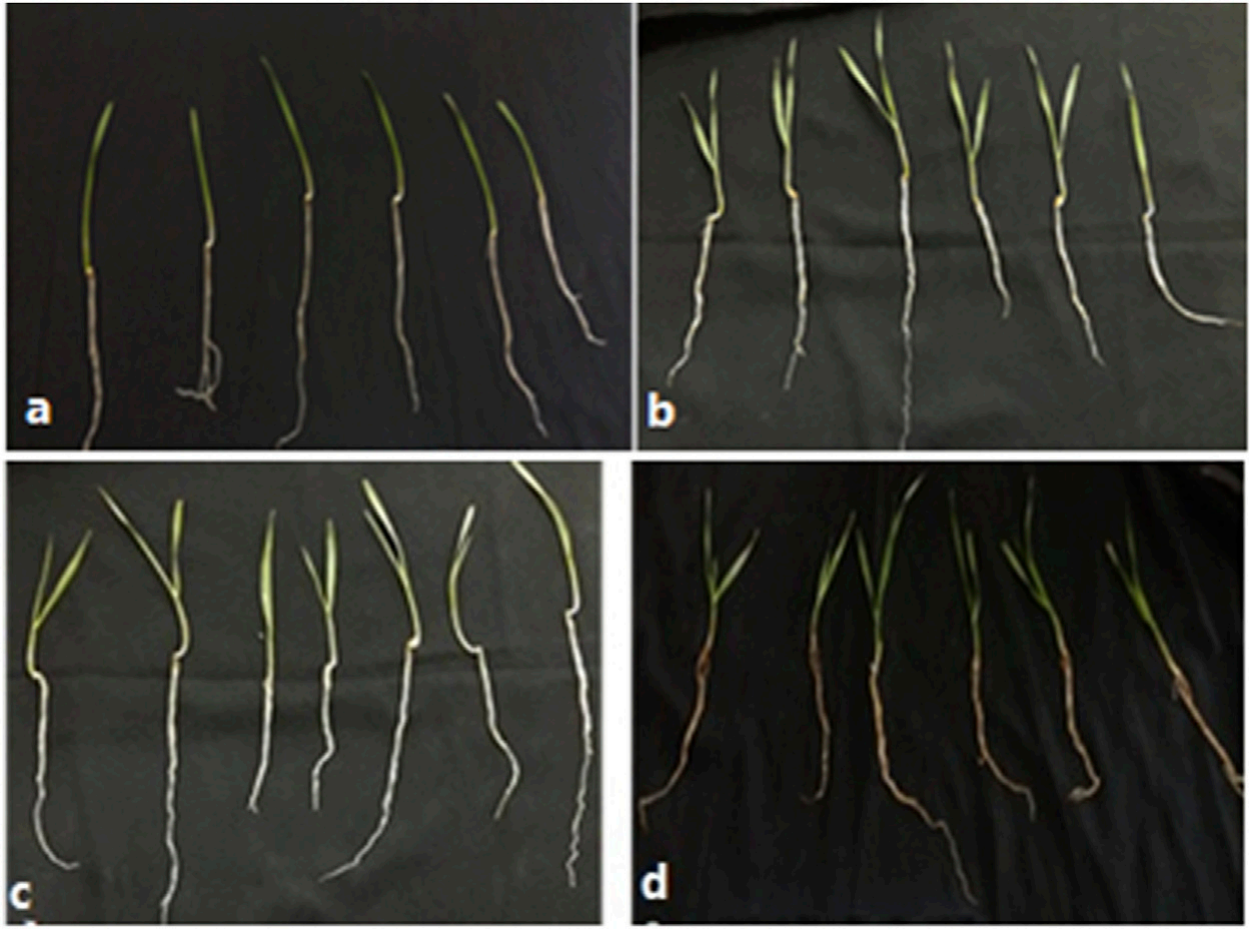
Effect of drought stress on leaf pigmentation: **(A)** control-1 plants [23(0)]; **(B)** drought-stressed plants [23(7)]; **(C)** control-2 plants [31(0)]; **(D)** post-drought recovery plants [31(7)].

The leaves of the wheat in the control plants were green in color. The leaves turned to pale yellow when they were exposed to drought stress. After recovery from drought stress, the leaves’ color changed to light green (Figure 2).

### Effect of drought stress on wheat shoot length and weight and its post-drought recovery

**FIGURE 3:**
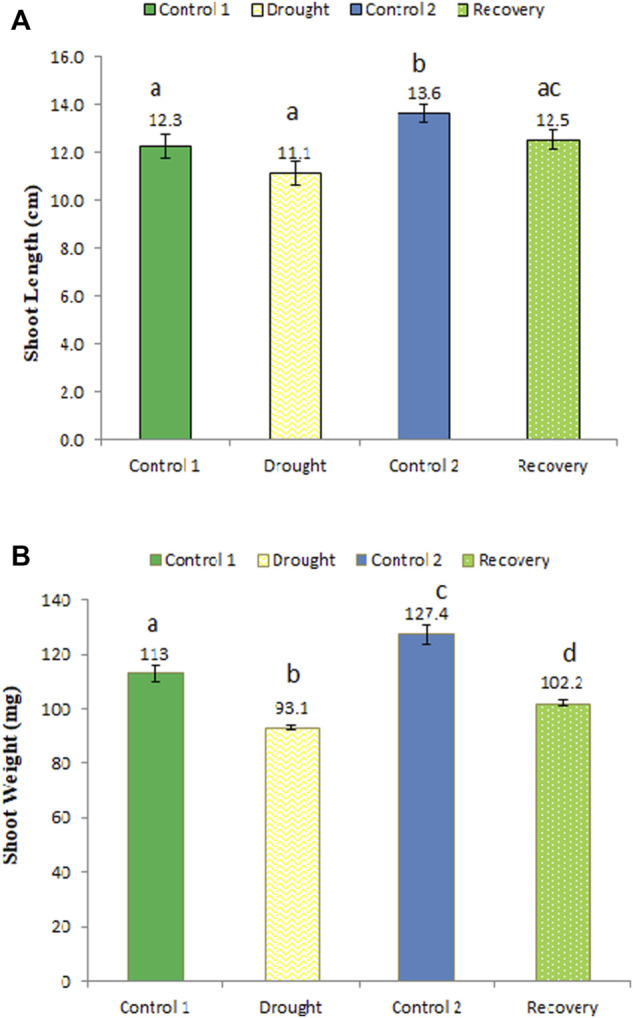
Effect of drought stress on the shoot length **(A)** and weight **(B)** of wheat and its recovery after drought stress. Length is measured in centimeters and weight in milligrams. The data represent the mean of three independent replications. Different letters above the column indicate significant changes when determined through Tukey’s HSD test.

The shoot length was shorter after the 7-day drought stress (11.1 cm) compared to the control plants (12.3 cm), although this decrease of 10.81% was not statistically significant (Figure 3A). After the 7-day recovery, the shoot length increased to 12.5 cm, an increase of 12.61%, which represents a significant recovery compared to drought-stressed stage. Similarly, shoot weight was significantly lower under drought stress (93.1 mg) compared to the same-aged control plants’ shoot weight of 113 mg, i.e., a decrease of 21.38% (Figure 3B). After the 7-day recovery, the shoot weight increased considerably to 102.2 mg (an increase of 9.77%).

### Effect of drought stress on wheat root length and weight and its post-drought recovery

**FIGURE 4:**
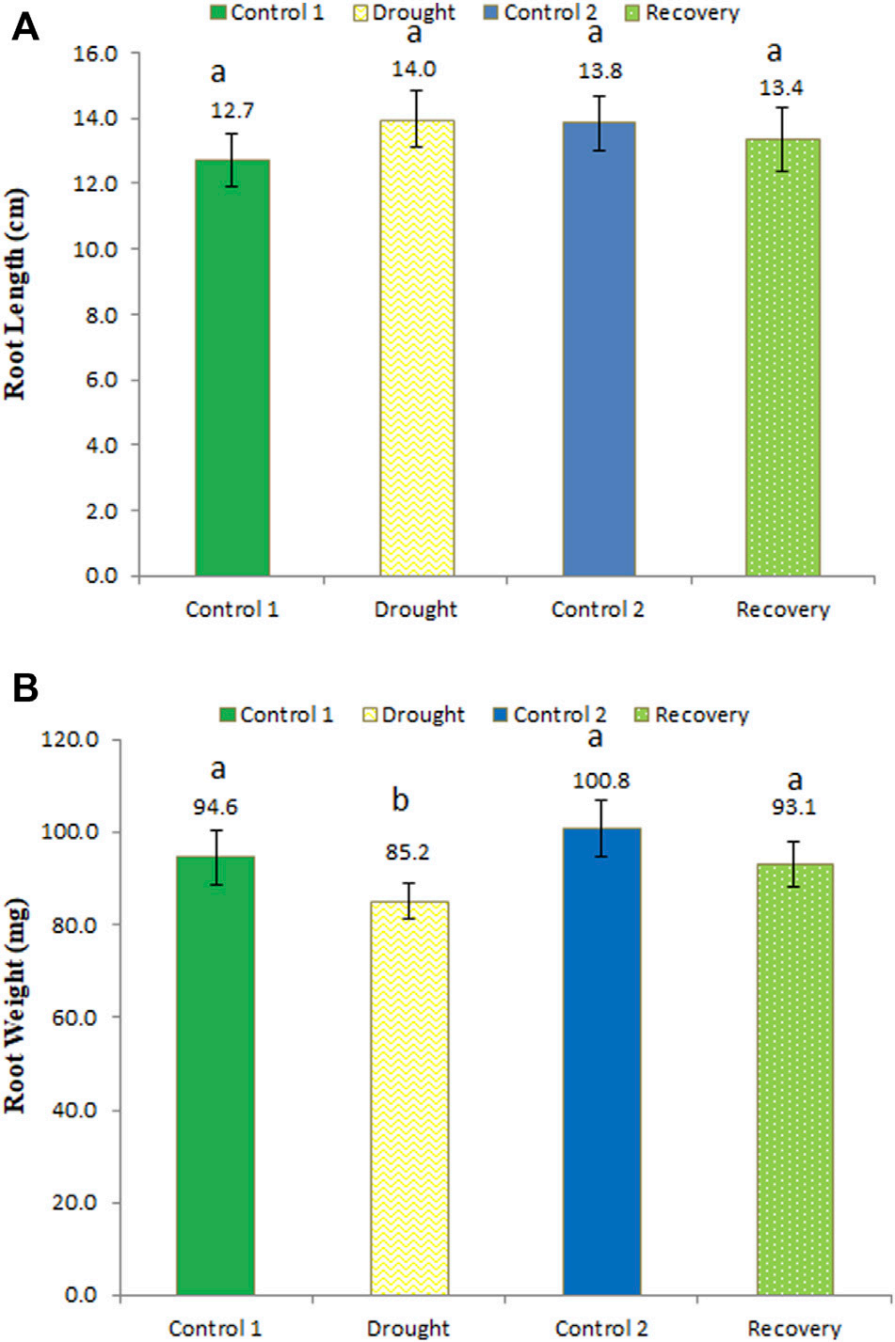
Effect of drought stress on the root length **(A)** and weight **(B)** of wheat and its recovery after drought stress. Length is measured in centimeters and weight in milligrams. The data represent the mean of three independent replications. Different letters above the column indicate significant changes when determined through Tukey’s HSD test.

An longer root length (14 cm) was recorded under drought stress compared to the same-aged control plants (12.7 cm) (Figure 4A) (an increase of 10.24%). After the 7-day recovery, the root length observed was 13.4 cm, which was restored and normalized to the control plants. These changes in root length were not statistically significant. Drought stress significantly reduced root weight to 85.2 mg compared to the control plants of the same age (94.6 mg) (a 9.94% decrease in weight), while in the recovery stage, the root weight significantly increased to 93.1 mg (an increase of 9.27%) (Figure 4B).

### Effect of drought stress on ascorbate peroxidase activity in wheat

**FIGURE 5:**
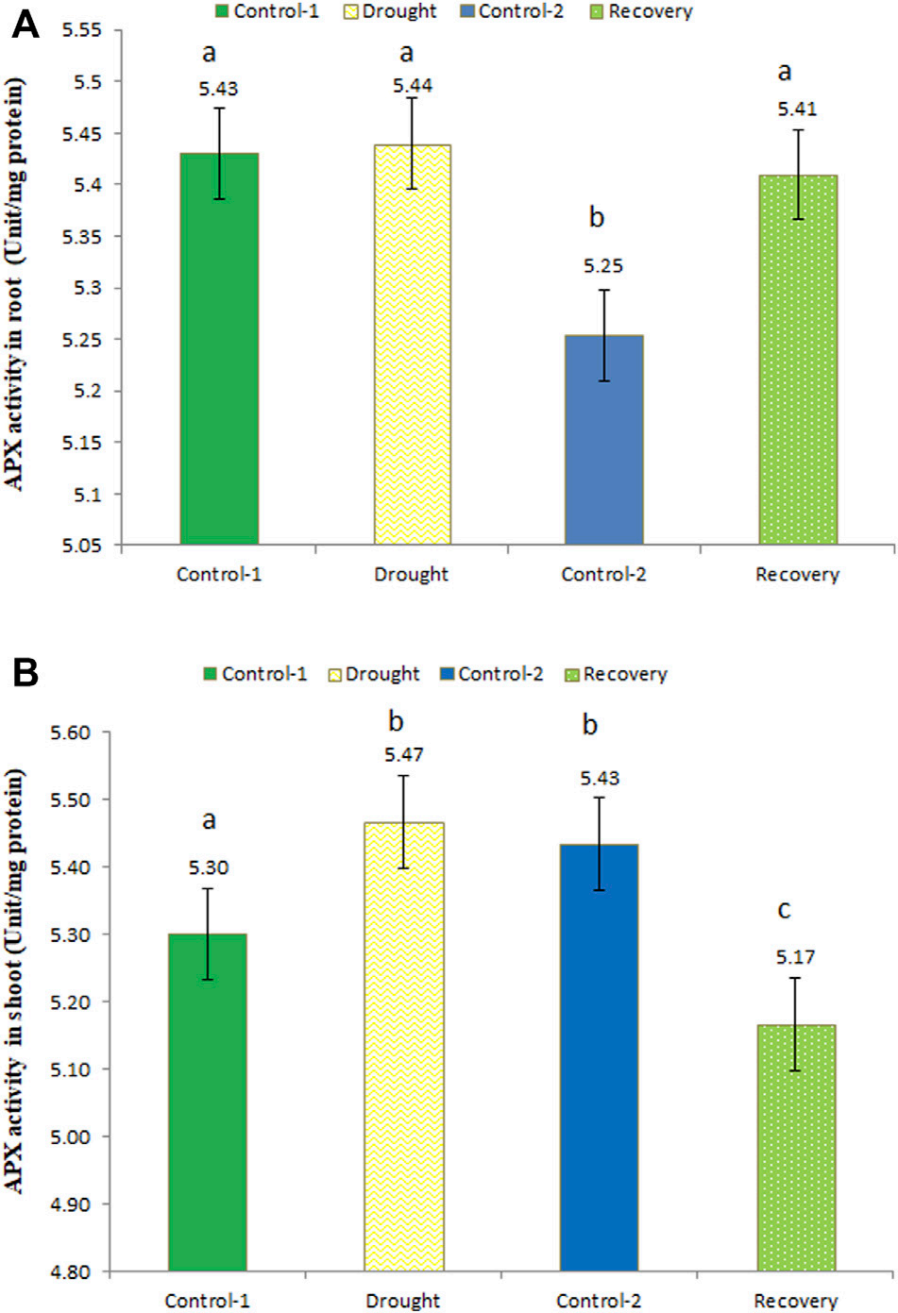
Enzyme activity of ascorbate peroxidase (APX) in the roots **(A)** and shoots **(B)** of wheat under control, drought, and recovery stages. The data represent the mean of three independent replications. Different letters above the column indicate significant changes when determined through Tukey’s HSD test.

Ascorbate peroxidase activity was determined at different time points, i.e., under control, drought, and recovery conditions, in the wheat roots. After the 7-day drought, APX activity was slightly increased. APX activity under drought stress was 5.44 unit/mg protein, while in the recovery phase the enzyme activity was a little lower (5.41 unit/mg protein) (Figure 5A). APX activity at the recovery stage was significantly higher compared to age-matched control-2 plants, whose activity was recorded as 5.25 unit/mg protein.

In drought-stressed wheat shoots, APX activity was higher compared to the control (5.47 unit/mg protein; an increase of 3.21%), while in recovery stage, the activity was lower (5.17 unit/mg protein) (Figure 5B). APX activity changes b/w for age-matched control-1 and drought-stressed as well as control-2 and post-drought recovery plants were statistically significant in wheat shoots.

### Effect of drought stress on fatty acids profile

Fatty acid composition was detected in the shoots of the wheat cultivar at different time points, i.e., control, drought, and recovery conditions.

### Fatty acids percent composition changes in control, drought-stressed, and post-drought recovery wheat

Fatty acids were detected under control conditions. Control-1 was 16-day old plants, while control-2 was 31-day old plants. The main fatty acids detected in the control plants were palmitic acid (C16:0), stearic acid (C18:0), oleic acid (C18:1), linoleic acid (C18:2), linolenic acid (C18:3), and behenic acid (C22:0) (Table 1). The fatty acid which was detected in the highest amount in control-1 was linolenic acid (55.23%), while oleic acid was found in the lowest amount (0.95%). The same trend was followed in control-2 plants collected after 31 days of sowing, where the highest amount was linolenic acid (49.06%), while the fatty acid detected with the lowest amount was oleic acid (2.60%). Stearic and behenic acids were not detected (ND) in the control-2 plants.

**TABLE 1:** Fatty acid percent composition in wheat samples at different stages.

Fatty acid	Retention time	Control-1	Drought-stressed	Control-2	Post-drought recovery
Lauric acid (C12:0)	16.93	ND	ND	ND	55.42
Myristic acid (C14:0)	20.87	ND	ND	ND	0.13
Palmitic acid (C16:0)	25.52	33.71	39.91	42.93	23.25
Stearic acid (C18:0)	31.43	1.44	ND	ND	1.50
Oleic acid (C18:1)	31.95	0.95	2.11	2.60	1.57
Linoleic acid (C18:2)	33.45	7.52	6.85	5.41	7.01
Linolenic acid (C18:3)	35.80	55.23	51.18	49.06	40.08
Behenic acid (C22:0)	43.35	1.14	ND	ND	ND

*ND: Not Detected.

The fatty acid composition changed when analyzed at the end of the 7-day drought stress compared to the age-matched control plants. The main fatty acids detected under drought conditions were palmitic acid, oleic acid, linoleic acid, and linolenic acid. During stress conditions, the linolenic acid was observed with the highest quantity (51.18%), while oleic acid was detected with the lowest amount (2.11%). The results revealed 39.91% palmitic acid and 6.85% linoleic acid in the drought-stressed plants (Table 1).

The fatty acid percent composition was analyzed at the end of the 7-day recovery period. The main fatty acids detected under the recovery stage were lauric acid, myristic acid, palmitic acid, stearic acid, oleic acid, linoleic acid, and linolenic acid. In the post-drought recovery plants, lauric acid was quantified as the highest (55.42%), while the acid with the lowest quantity was myristic acid (0.13%) (Table 1). The amounts of palmitic acid (23.25%) and linolenic acid (40.08%) were lower compared to the control plants of the same age.

### Percent compositions of fatty acids commonly detected in control, drought-stressed, and recovery wheat

**FIGURE 6:**
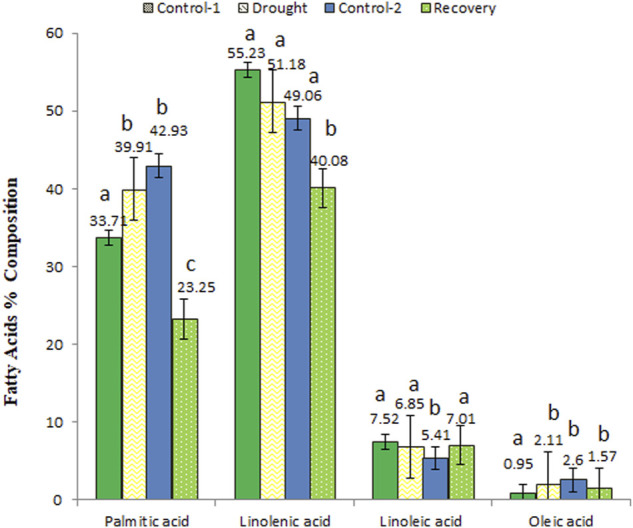
The variations in percent composition of common fatty acids detected in control, drought-stressed, and post-drought recovery plants. Different letters above the column indicate significant changes when determined through Tukey’s HSD test.

Palmitic acid, linolenic acid, linoleic acid, and oleic acid were commonly identified and quantified at all analyzed time points (Figure 6). The quantities of palmitic acid (from 33.71 to 39.91%) and oleic acid (from 0.95 to 2.11%) increased under drought stress but decreased when analyzed at the end of post-drought recovery stage compared to age-matched control plants. On the other hand, slight changes in linoleic acid and linolenic acid concentrations were observed among different analyzed stages.

## Discussion

Drought is the major stress factor restricting reproductive growth, plant development, and basically survival (Flexas and Medrano, 2002). More than 70% of fertile land around the globe is affected by drought, and the yield loss related to drought stress has gained much attention in recent years as the activities of agriculture have been increased to less arable-friendly or more infertile lands to meet the requirements for growing food (Budak et al., 2013). The present study was executed to test the effect of drought stress and post-drought recovery on the morphological, enzymatic, and metabolite levels of the selected wheat variety, Atta Habib.

The current study has shown that the leaves of the wheat cultivar Atta Habib in the control plants were green in color. The leaves turned to pale yellow when they were exposed to drought stress. After recovery from drought stress, the leaves’ color changed to light green. Similar results were reported by Zaharieva et al. (2001), who asserted that the pale yellow color is likely to be a passive adaptation to drought-stress conditions as the green color of *Aegilops geniculata* was affected by drought stress and changed to pale yellow. Our results are also in agreement with Azenas et al. (2018), who studied the effect of water-limiting conditions on the leaf color of five Mediterranean species. The present and previous studies have suggested changes in plant pigmentation and color under water-limiting/drought conditions. The shoot and root weight and shoot length decreased when the wheat was exposed to drought stress for 7 days, although they recovered to some extent after the 7-day recovery phase, while the root length increased during the drought period as they searched for water. Similar findings were reported in soybean (Khan and Komatsu, 2016), in which the root length of soybean increased after 4 days of drought conditions, while recovering to normal after 4 days of recovery, whereas root weight decreased after exposure to stress, while in the recovery stage the root weight increased. The results of the current study are also in agreement with the study of Jaleel et al. (2009), in which the growth of roots and shoots was affected by drought stress.

In the present study, APX activity increased in the wheat under drought-stress conditions, while it decreased in the recovery phase. These results confirm the findings of Hameed et al. (2011), in which different wheat genotypes showed elevated APX activities under drought stress as a tool to minimize oxidative damage. Wang et al. (2016) reported an increased level of APX activity in the shoots of Alfalfa under drought conditions. Increased APX activity was also revealed in drought-stressed soybean (Kausar et al., 2012). These findings suggest the crucial role of APX in scavenging peroxides as an effort to reduce/repair the oxidative damage caused by drought, and when the plant moves towards the post-drought recovery phase, the enzyme activity is normalized.

The current study has shown that the fatty acid composition of Atta Habib is affected under drought stress and during post-drought recovery conditions. In the present study, the percent composition of palmitic acid and oleic acid increased under drought conditions, while linoleic acid and linolenic acid decreased under stress. In the recovery phase, the percent composition of palmitic and oleic acid decreased, while linoleic and linoleic acid increased. Our results are in agreement with those of Laribi et al. (2009), who reported an increase in the quantities of palmitic acid and oleic acid under drought stress, while a reduction was observed in the levels of linoleic and linolenic acid. Similar results were reported by Baldini et al. (2002), who suggested that drought stress caused accelerated and earlier embryo development and stimulated the enzymatic activities of fatty acid biosynthesis due to which elevated levels of fatty acids were observed under drought-stress conditions. In a more recent study on safflower genotypes (Joshan et al., 2019), drought induced an increase in palmitic, stearic, and oleic acids. Palmitic acid either forms background storage fats and oils or the hydrophobic matrix of cell membranes and the components of cuticle waxes. Palmitic acid is the primary fatty acid formed in the cell that gives rise to higher fatty acids by modifications such as the elongation, desaturation, and insertion of various functional groups (Sidorov et al., 2014). Oleic acid stimulates signaling enzyme phospholipase D that has an anti-cell death function (Zhang et al., 2003). Oleic acid also regulates levels of nitric-oxide-associated protein, thus regulating nitric-oxide-mediated defense signaling in Arabidopsis (Mandal et al., 2012). Drought stress increases the fatty acid saturation of plasma membrane lipids, which leads to membrane rigidification (Lopez-Perez et al., 2009). Saturated fatty acids are involved in shifting membrane fluidity under adverse environmental conditions. Keeping in view the diverse functions of palmitic acid and oleic acid, the rise in their quantities accounts for their involvement in membrane fluidity as well as the synthesis of complex lipids as a way to cope with the stress.

Abiotic stresses have been shown to significantly decrease the amount of linoleic and linolenic fatty acids, while the amount of palmitic and oleic acids increases, suggesting that the plant membrane unsaturation decreases upon suffering stresses (Singer et al., 2016). The study reported that the decrease of membrane lipids in response to different abiotic stresses was mainly due the critical decrease in mono-galactosyldiacyl-glycerol content, compared with the reduction in di-galactosyldiacyl-glycerol and phosphatidyl-glycerol. The reduction in mono-galactosyldiacyl-glycerol content has been considered as a common adaptation strategy for plants when coping with drought, salinity, low temperature, and aluminum stresses (Wang et al., 2016). The degree of fatty acid desaturation has been found to decrease in plants under drought stress (Upchurch, 2008). The signaling roles of lipids or the intermediates of lipid biosynthesis and metabolism have been shown to play a crucial role in plants’ environmental stress response (Hou et al., 2016). Linoleic acid and linolenic acids have also been shown to be oxidized spontaneously or during the stress response, resulting in the synthesis of important signaling molecules, such as jasmonic acid, etc., that are involved in multiple signaling pathways (Savchenko et al., 2014). Linolenic acid has been found to participate in the synthesis of jasmonic acid during seedling drought and to alleviate drought stress through jasmonic acid signaling. Maize has been shown to tend to use free fatty acids as antioxidant substances to reduce drought-induced damage (Zi et al., 2022). Linolenic acid is also part of photosynthetic membranes, along with other cellular membranes. Non-tolerant plants subjected to drought and salt stresses commonly show decreased levels of linolenic acid in their membranes, which suggests that the decrease in linolenic acid points to the damage caused by the stress (Liu et al., 2019). In safflower, linoleic acid content has been found to decrease under drought stress (Joshan et al., 2019). Based on the results of current study, it was found that the abundances of palmitic acid and oleic acid increased, while linoleic acid and linolenic acid decreased under drought stress, indicating that the unsaturation of membranes decreases under drought stress. Similarly, unsaturation increased during the post-drought recovery period to restore the integrity of the membranes to enable normal cell functioning.

## Conclusion

It can be concluded from the results of the study that drought stress has a negative impact on morphological parameters such as root weight, shoot weight, and shoot length, while they tend to recover in the post-drought recovery wheat in the post-drought stage. The activity of ascorbate peroxidase increased in the drought-stressed plants, while it decreased in the recovery phase. Predominantly, palmitic acid and oleic acid increased, while linoleic and linolenic acid decreased in the drought-stressed plants, whereas in recovery phase, palmitic and oleic acid decreased. These results suggest that wheat copes with the oxidative damage induced by drought stress through scavenging toxic oxides through increased APX activity and adjusts fatty acid abundances in an effort to maintain membranes’ integrity and a suitable fat metabolism route, thus helping in drought response and recovery in the post-drought phase. As palmitic, oleic, linoleic, and linolenic aids have physiologically diverse roles in normal growth and development, as well as stress responses through increasing or decreasing membranes’ unsaturation and transport across, these fats’ metabolic re-adjustments under drought stress and during the post-drought recovery are beneficial for plants’ survival. The findings of the current study are important in revealing the drought stress and post-drought recovery mechanism and related adjustments in the fatty acid metabolism. Post-drought recovery is a relatively little studied area, and this study will provide a helpful basis to explore this avenue further.

## Data Availability

The original contributions presented in the study are included in the article/Supplementary material; further inquiries can be directed to the corresponding author.
